# A Symmetric Form of the Clausius Statement of the Second Law of Thermodynamics

**DOI:** 10.3390/e26060514

**Published:** 2024-06-14

**Authors:** Ti-Wei Xue, Tian Zhao, Zeng-Yuan Guo

**Affiliations:** 1 Key Laboratory for Thermal Science and Power Engineering of Ministry of Education, Department of Engineering Mechanics, Tsinghua University, Beijing 100084, China; xuetiwei@tsinghua.edu.cn (T.-W.X.); gszhaotian@126.com (T.Z.); 2 School of Energy Storage Science and Engineering, North China University of Technology, Beijing 100144, China

**Keywords:** the second law of thermodynamics, Clausius’s statement, theorem of equivalence of transformations, symmetry

## Abstract

Bridgman once reflected on thermodynamics that the laws of thermodynamics were formulated in their present form by the great founders of thermodynamics, Kelvin and Clausius, before all the essential physical facts were in, and there has been no adequate reexamination of the fundamentals since. Thermodynamics still has unknown possibilities waiting to be explored. This paper begins with a brief review of Clausius’s work on the second law of thermodynamics and a reassessment of the content of Clausius’s statement. The review tells that what Clausius originally referred to as the second law of thermodynamics was, in fact, the theorem of equivalence of transformations (TET) in a reversible cycle. On this basis, a new symmetric form of Clausius’s TET is proposed. This theorem says that the two transformations, i.e., the transformation of heat to work and the transformation of work from high pressure to low pressure, should be equivalent in a reversible work-to-heat cycle. New thermodynamic cyclic laws are developed on the basis of the cycle with two work reservoirs (two pressures), which enriches the fundamental of the second law of thermodynamics.

## 1. Introduction

As with the first law of thermodynamics, the second law of thermodynamics is accepted by physicists as perhaps the most secure generalization from experience that we have [1]. It is generally believed that the second law of thermodynamics is a rule about process irreversibility, and along with that, entropy generation is an important physical quantity for quantifying it. Clausius [2,3] once gave a famous statement about the second law of thermodynamics, which says that “*heat can never pass from a cold to a warm body without some other change, connected therewith, occurring at the same time*”. It is an empirical summing-up of the tendency of the irreversible heat transfer phenomenon [4].

Nonetheless, Xue et al. [4,5,6] reviewed the history of Clausius’s establishment of thermodynamics in the mid-19^th^ century to find that, in fact, the second law of thermodynamics claimed by Clausius has nothing to do with irreversibility. Clausius incorporated the two laws of thermodynamics into a symmetrical theoretical framework, where he regarded the theorem of equivalence between heat and work as the first law and the theorem of equivalence of “*transformations*” (TET) as the second law and considered them to be of the same kind [2,3]. The TET was proposed in the context of a reversible thermodynamic cycle [3,7,8]. Moreover, the concept of entropy was proposed based on the TET, which is why entropy is required to be defined under reversible conditions. Prigogine [9] once pointed out that thermodynamics throughout the nineteenth century remained in the ideal reversible conversion stage and did not involve the theory of entropy of irreversible processes. The origin of the second law of thermodynamics was not accurately understood due to the fact that history was always reconstructed, as a matter of course, by those who came after it. Moreover, the founders of thermodynamics relied only on the facts available to them at the time, leading to the fundamentals of the second law of thermodynamics possibly being incomplete. Consequently, thermodynamics was susceptible to logical insecurity [1].

In view of the above concerns, it is necessary to re-examine the fundamentals of the second law of thermodynamics. In Clausius’s work, the statement that *heat can never pass from a cold to a warm body without some other change* was actually regarded merely as a fundamental principle to prove his TET. After Clausius, von Oettingen, a scientist who advocated for thermodynamic symmetry, came up with a statement similar to Clausius’s fundamental principle, *volumetric work can never pass from low pressure to high pressure without other change* [10]. Based on the statement by von Oettingen, this paper proposes a new symmetric form of Clausius’s TET, which is a supplement to the fundamentals of thermodynamics.

## 2. The Second Law of Thermodynamics Claimed by Clausius

**Figure 1:**
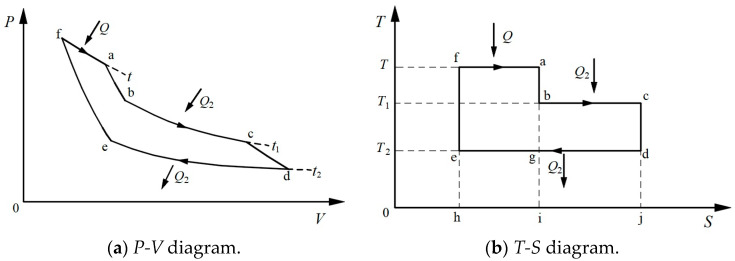
The thermodynamic cycle with three heat reservoirs as proposed by Clausius [4].

Clausius’s motivation for developing the second law of thermodynamics was to determine the Carnot efficiency. Although Carnot presented the famous Carnot’s theorem as early as 1824, due to a misunderstanding of heat at the time (the caloric theory of heat), Carnot did not obtain an exact expression for Carnot’s efficiency. After the theorem of equivalence between heat and work was established, Clausius was aware that there were two kinds of transformations, i.e., the transformation of work to heat and the transformation of heat at high temperatures to low temperatures in reversible thermodynamic cycles [2,3]. Clausius considered that Carnot’s theorem expressed an equivalent relation between these two kinds of transformations. He called it the theorem of equivalence of transformations (TET). Clausius further elaborated this theorem by means of a thermodynamic cycle with three heat reservoirs, as shown in Figure 1a [4]. The concept of absolute temperature was not yet established at that time, and so the temperature was denoted by “*t*”. With the concept of absolute temperature later on, a more intuitive representation of this three-heat-reservoir cycle could be made using a *T-S* diagram, as shown in Figure 1b. Clausius assigned “*equivalence-values*” to the transformation of work to heat as *Q f* (*t*), corresponding to the inverse of process f–a, and the transformation of heat from high temperature to low temperature as *Q*_2_ *F*(*t*_1_, *t*_2_), corresponding to a combination of the processes b–c and d–e, where *f* (*t*) is a function of the temperature *t* only and *F*(*t*_1_, *t*_2_) is a function of the temperatures *t*_1_ and *t*_2_. With the help of the basic principle that *heat can never pass from a colder to a warmer body without some other change*, Clausius argued that the sum of the equivalence-values for all transformations in the reversible three-heat-reservoir cycle is equal to zero, as follows:

(1) $$-Qf\left(t\right)+Q_{2}F\left(t_{1},t_{2}\right)=0.$$

Clausius proved that there is a relation between the two functions, as follows:

(2) $$F\left(t_{1},t_{2}\right)=f\left(t_{2}\right)-f\left(t_{1}\right),$$

and the integration factor, *f* (*t*), was exactly the reciprocal of the absolute temperature, 1/*T*, as follows:

(3) $$f\left(t\right)=\frac{1}{T}.$$

Thus, for a general reversible thermodynamic cycle with multiple heat sources and sinks, Equation (1) can be rewritten as follows:

(4) $$\oint \frac{1}{T}\delta Q=0.$$

Equation (4) was referred to as the mathematic expression of the TET by Clausius (later named the Clausius equality). Based on the TET, Clausius confirmed the expression for Carnot efficiency, *η*_C_, as follows:

(5) $$\eta_{C}=1-\frac{T_{2}}{T_{1}}.$$

Clausius succeeded in unravelling the mystery that Carnot had left unsolved for 30 years. Equation (4) implies the existence of a state variable. It was not until 1865 that Clausius officially named it entropy, *S*, as follows:

(6) $$dS=\frac{\delta Q_{\mathrm{rev}}}{T},$$

where the subscript, rev, denotes reversibility.

Clausius’s main conclusions about the second law of thermodynamics came from the analysis of reversible cycles. Irreversibility is, of course, an existence not to be ignored. Clausius used the idea of compensating for transformations to explain the differences between reversibility and irreversibility [2,3]. In a reversible cyclic process, these two transformations compensate for each other and, correspondingly, the equivalence-values of the two transformations cancel each other out, as expressed by the Clausius equality, Equation (4). On the contrary, for the irreversible process in which heat is generated by friction, only a work-to-heat transformation exists. Unlike reversible processes, the transformation for this irreversible process is not compensated by the transformation of heat from low to high temperatures. Similarly, the irreversible heat transfer process due to a temperature difference has only the transformation of heat from high to low temperatures, which is likewise uncompensated. Clausius referred to the transformations involving irreversibility as “*uncompensated transformations*” [2,3]. As a result, the algebraic sum of the equivalence-values of all transformations in an irreversible thermodynamic cycle can only be negative [2,3], as follows:

(7) $$\oint \frac{1}{T}\delta Q<0.$$

Equation (7) was called the Clausius inequality. Furthermore, the entropy in an irreversible heat transfer process has the following relation:

(8) $$dS>\frac{\delta Q}{T}.$$

The principle of entropy increase for isolated systems was summarized based on Equation (8). Since then, the second law of thermodynamics has been widely recognized as the law governing the irreversibility of processes [11,12,13,14,15]. Correspondingly, the summing-up of a natural phenomenon, “heat can never pass from a cold to a warm body without some other change”, has been considered as the Clausius statement of the second law of thermodynamics. However, Clausius himself actually regarded the TET for reversible thermodynamic cycles as the second law of thermodynamics, which was explicitly mentioned in his memoirs [2]. The summing-up plays a role as a fundamental principle to demonstrate the TET in Clausius’s work. The former had its roots in experience alone and had no other support, except that it was consistent with the first law [16,17]. The latter has a clear mathematical expression and acts as a more solid logical basis for the second law of thermodynamics [4]. Although, as Leff [18] said, the idea of the equivalence of transformations was difficult to grasp, and therefore, it was not even mentioned in most thermodynamic textbooks, though once it is mastered, it might be possible to help to understand and develop thermodynamics fundamentally. Thermodynamics in the 19th century focused on equilibrium and reversible processes, with the discussion of irreversibility taking a back seat [19]. The effects of irreversibility were only formulated as corollaries of the TET.

## 3. Another Statement of the Second Law of Thermodynamics

Thermodynamics, as Bridgman [19] said, is still an unfinished subject, or at least one whose ultimate possibilities have not yet been explored. Symmetry, as a powerful tool of thought, greatly contributed to the development of twentieth-century physics. However, it was absent from the crucial period of the nineteenth century in which thermodynamics was created. Therefore, it is necessary to re-examine the fundamentals of thermodynamics based on the idea of symmetry, whereby new aspects can be expected to be discovered.

### 3.1. Thermodynamic Symmetry

**Figure 2:**
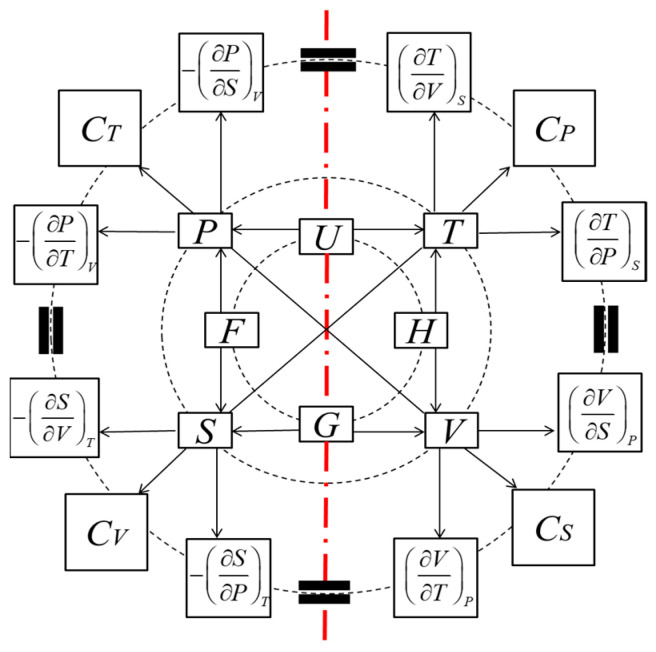
The thermodynamic wheel of connections (TWC) [21]. The directions of the arrows suggest the partial derivations.

Thermodynamics contain rich symmetries. As early as 1885, von Oettingen [20] proposed a symmetric framework for thermodynamics. He pointed out that there is a physical and linguistic symmetry between thermal and mechanical variables and functions [6]. The thermodynamic wheel of connections [21] can be used to express this symmetric framework, as shown in Figure 2. For each pair of relations, a pair of symmetric ones can arise from symmetric transformations expressed as exchanges of variables, *T* ↔ *P* and *S* ↔ −*V*. From a typographical point of view, this complementarity leads to two columns of exposition. 

Von Oettingen further compared the formula for calculating volumetric work in quasi-static processes with Equation (6), as follows:

(9) $$dV=-\frac{\delta W_{\mathrm{rev}}}{P},$$

and he argued that there is a certain symmetry between the two (in fact, Maxwell [22] had also done so before this). Analogous to Clausius’s fundamental principle that *heat can never pass from a cold to a warm body without some other change*, he provided a symmetric statement, i.e., “*volumetric work can never pass from low pressure to high pressure without other change*” [10]. Since Clausius developed the TET based on his fundamental principle, it is possible to develop a symmetric TET based on von Oettingen’s symmetric fundamental principle.

### 3.2. Symmetric Form of the Carnot Theorem

**Figure 3:**
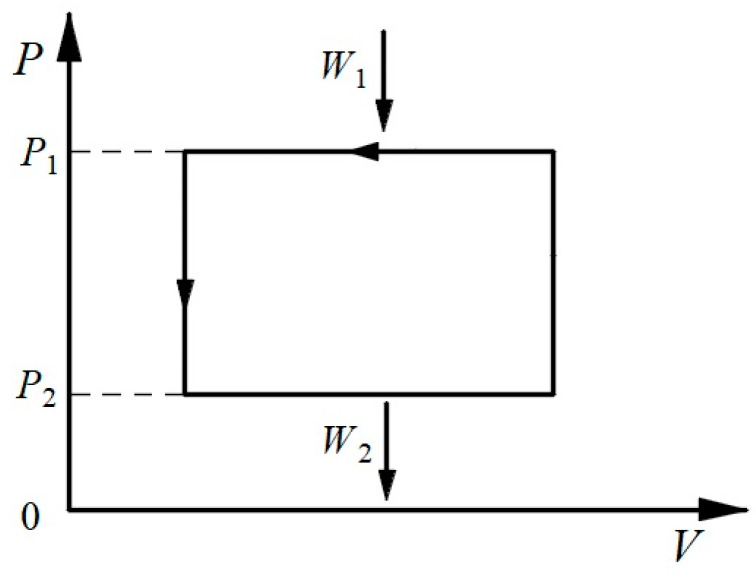
The isobaric-isochoric cycle.

The Carnot cycle consists of two isothermal and two isentropic processes. Based on the symmetry von Oettingen referred to, it is straightforward to surmise that a cycle symmetrical to the Carnot cycle should contain two isobaric and two isochoric processes, and this is referred to here as an “*isobaric-isochoric cycle*” (or a rectangular cycle, as mentioned in some references [23,24,25]), as shown in Figure 3. As we know, the ideal Carnot cycle operates through reversible heat exchanges with two heat reservoirs and reversible work exchanges with an infinite number of work reservoirs. Here, the ideal isobaric-isochoric cycle operates through reversible work exchanges with two work reservoirs and reversible heat exchanges with an infinite number of heat reservoirs.

**Figure 4:**
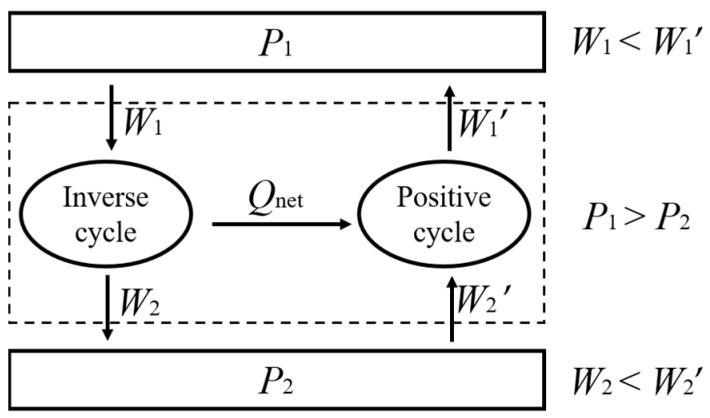
The combined cycle consisting of two reversible heat pumps.

Similar to the heat efficiency of a positive (heat-to-work) cycle, the work efficiency of an inverse (work-to-heat) cycle, *η_W_*, can be defined as the ratio of the net heat output through the cycle to the work input from the high-pressure work reservoir, as follows:

(10) $$\eta_{W}=\frac{Q_{\mathrm{net}}}{W_{\mathrm{in}}},$$

where the subscript, net, denotes a net quantity. Similar to net work, net heat is also a manifestation of the converted energy in a thermodynamic cycle and is quantitatively equal to net work. The Carnot theorem states that the heat efficiency of a reversible Carnot cycle is only dependent on the temperatures of the two heat reservoirs and not on the nature of the cyclic working fluid. Similarly, it can be demonstrated by counterargument that the work efficiency of a reversible isobaric-isochoric cycle is only related to the pressures of the two work reservoirs, as follows:

(11) $$\eta_{W}=1-\frac{W_{2}}{W_{1}}=\Phi \left(P_{1},P_{2}\right),$$

where Φ(*P*_1_, *P*_2_) is a function of the pressures of two work reservoirs. It is important to emphasize that the work discussed here is the volumetric work transferred due to a pressure differential without the help of external components such as leverage or gears. Mechanical work transferred through a lever mechanism, although unavoidable in engineering applications, will cause a change in the state of the external components, which is contrary to the idea of “without other change”. We can conceive a combined cycle consisting of two reversible heat pumps between the same high- and low-pressure work reservoirs, as shown in Figure 4. If we assume that the two heat pumps have different work efficiencies due to the employment of different working fluids, then we can let the reversible heat pump with a higher work efficiency produce heat through an inverse cycle, and then we can feed all the net heat produced into another heat pump to produce work through a positive cycle. Since the latter heat pump operating in a positive cycle has a lower work efficiency, it needs to absorb more work from the low-pressure work reservoir compared to the work released to the low-pressure work reservoir by the former heat pump, and accordingly, the latter heat pump releases more work to the high-pressure work reservoir compared to the work absorbed from the high-pressure work reservoir by the former heat pump. Thus, the total effect in the combined cycle is that the low-pressure work reservoir transfers work to the high-pressure work reservoir without other change, which is inconsistent with the fundamental principle stated by von Oettingen. Therefore, the above assumption is not valid, and the two reversible heat pumps should have the same work efficiency between the same high- and low-pressure work reservoirs. Equation (11) is thus proven. As such, a theorem symmetrical to Clausius’s TET can be established to determine the specific expression for the work efficiency of reversible isobaric-isochoric cycles.

### 3.3. Symmetrical Form of Clausius’s TET

**Figure 5:**
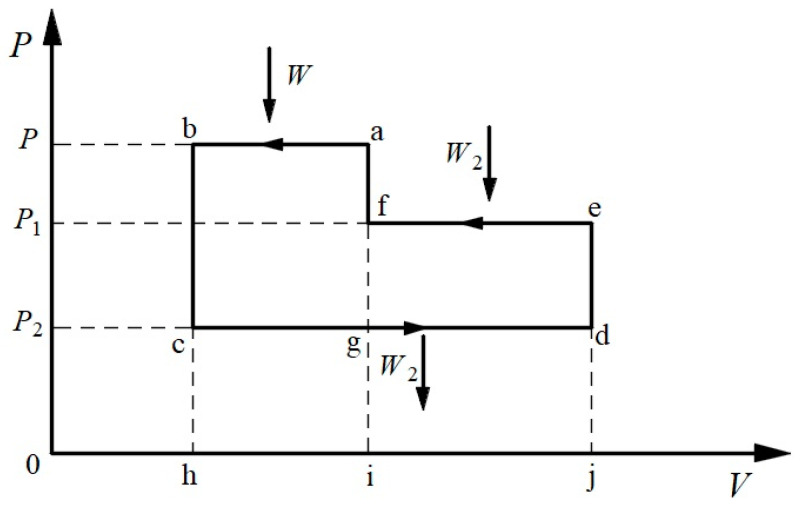
The thermodynamic cycle with three work reservoirs.

Similar to Clausius’s TET, the two transformations—the transformation of heat to work and the transformation of work from high pressure to low pressure—should be equivalent in a reversible work-to-heat cycle. Further analogous to Clausius’s three-heat-reservoir reversible positive cycle, the above symmetric TET can be quantified by constructing a three-work-reservoir reversible inverse cycle. It consists of three isobaric processes and three isochoric processes, as shown in Figure 5, where processes a–b, e–f, and c–d are isobaric processes with the pressures, *P*, *P*_1_, and *P*_2_, and the works exchanged, *W*, *W*_2_, and *W*_2_, respectively. All other processes are isochoric processes.

Since the input work, *W*_2_, of process e–f is equal to the output work, *W*_2_, of process c–d, the combination of processes e–f and c–d can be regarded as the transformation of work, *W*_2_, from high pressure to low pressure, and accordingly, process a–b is regarded as the transformation of work to heat. If the equivalence-value of the transformation of work from high pressure to low pressure is regarded as a positive value, then the equivalence-value of the transformation from heat to work is positive and the reverse is negative. For the transformation of heat to work, corresponding to the inverse of process a–b, its equivalence-value is proportional to the amount of work, *W*, and it depends on the pressure, *P*, which is expressed as follows:

$$Wg\left(P\right),$$

where *g*(*P*) is a function of the pressure, *P*, only. For the transformation of work from high pressure to low pressure, corresponding to the combination of processes c–d and e–f, its equivalence-value is proportional to the amount of work, *W*_2_, and it also depends on the two pressures, *P*_1_ and *P*_2_, which are expressed as follows:

$$W_{2}G\left(P_{1},P_{2}\right),$$

where *G*(*P*_1_, *P*_2_) is a function of the pressures, *P*_1_ and *P*_2_. These two equivalence-values should be algebraically equal. Thus, the algebraic sum of the two equivalence-values in a reversible three-work-reservoir cycle is zero, as follows:

(12) $$-Wg\left(P\right)+W_{2}G\left(P_{1},P_{2}\right)=0.$$

Equation (12) is the mathematical expression of the symmetric TET, which shows that the effects of the transformation of work to heat and the transformation of work from high pressure to low pressure in a reversible cycle cancel each other out.

Considering again the cycle shown in Figure 3, if the pressure *P* becomes *P′*, then accordingly, *W* becomes *W′*, and we obtain the following:

(13) $$-W^{\prime }g\left(P^{\prime }\right)+W_{2}G\left(P_{1},P_{2}\right)=0.$$

If *P′* is greater than *P*, then the cycle containing *P* can be reversed, and then the two cycles containing *P′* and *P* can be superimposed as a new inverse isobaric-isochoric cycle, with the high-pressure work reservoir at *P′* and the low-pressure work reservoir at *P*. The following relation can then be obtained based on Equations (12) and (13):

(14) $$\frac{W}{W^{\prime }}=\frac{g\left(P^{\prime }\right)}{g\left(P\right)}.$$

In this inverse isobaric-isochoric cycle, *W′* can be regarded as containing two parts, *W*’-*W* and *W*. The first part is the amount of work in the work-to-heat transformation at *P′*, and the second part is the amount of work in the transformation from *P′* to *P*. Similar to Equation (12), the following relation can then be obtained:

(15) $$-\left(W^{\prime }-W\right)g\left(P^{\prime }\right)+WG\left(P^{\prime },P\right)=0.$$

Combining Equations (14) and (15), we obtain the following:

(16) $$G\left(P^{\prime },P\right)=g\left(P\right)-g\left(P^{\prime }\right).$$

Thus, the equivalence-value of the transformation of work between two pressures can be expressed as follows:

(17) $$WG\left(P_{1},P_{2}\right)=Wg\left(P_{2}\right)-Wg\left(P_{1}\right).$$

According to Equation (17), the transformation of work between two pressures can be understood as a combination of two opposite transformations between heat and work. Thus, in the case of continuous changes in pressure, the sum of the equivalence-values of all transformations in a reversible cycle is as follows:

(18) $$N=\oint g\left(P\right)\delta W=0.$$

Equation (18) is symmetric to the Clausius equality, Equation (4). Similarly, it implies the existence of a certain state variable. Substituting Equation (9) into Equation (18) yields the following:

(19) $$-\oint g\left(P\right)PdV=0.$$

When *g*(*P*) is equal to a negative reciprocal of pressure, we obtain the following:

(20) $$g\left(P\right)=-\frac{1}{P},$$

and Equation (19) becomes the following:

(21) ∮dV=0.

Therefore, the state variable implied in Equation (18) is none other than “*volume*”. The symmetric TET provides a new physical meaning for volume. The change of entropy expresses the equivalence-value of the transformations in Clausius’s TET, and so entropy was called “*the transformational content of the body*” [2]. In the new symmetric TET, the change of volume is used to express the equivalence-value of the transformations, and so volume could be understood as the transformational content of the body, similar to entropy. Von Oettingen treated entropy as an analogue of volume, and he called entropy “*heat volume*” [10]. Here, volume can be called “work entropy”, analogous to the concept of entropy. The concept of “work entropy” expresses the physical connotation of volume in the process (cycle) of a heat–work conversion. The symmetry between entropy and volume that we are currently discussing is restricted to reversible transformations. It is no longer satisfactory once irreversibility is involved. However, one thing to be aware of is that symmetry and asymmetry are the unity of opposites. Asymmetry in an irreversible case does not affect the validity of symmetry in a reversible case.

Thus, based on the theorem symmetrical to Clausius’s TET, it is sufficient to determine the work efficiency of a reversible inverse isobaric-isochoric cycle. Substituting Equations (14) and (20) into the expression for work efficiency yields the following:

(22) $$\eta_{W}=1-\frac{W_{2}}{W_{1}}=1-\frac{g\left(P_{1}\right)}{g\left(P_{2}\right)}=1-\frac{P_{2}}{P_{1}}.$$

Just as the temperature in the Carnot heat efficiency is the absolute temperature, the pressure in the work efficiency is the absolute pressure. It can be seen that there is good symmetry between the work efficiency of the reversible inverse isobaric-isochoric cycle, Equation (22), and the heat efficiency of the reversible Carnot cycle, Equation (5). Since the absolute zero of the temperature cannot be reached, the heat efficiency of the reversible Carnot cycle can only be less than one, which means that the amount of heat absorbed from the high-temperature heat reservoir cannot be fully converted into work. Similarly, since the absolute zero of the pressure (the absolute vacuum) cannot be reached, the work efficiency of the reversible inverse isobaric-isochoric cycle can only be less than one, and the amount of work input from the high-pressure work reservoir cannot be completely converted into heat. Of course, for irreversible processes, for example, with frictional heat loss or Joule heat loss, work can be fully converted into heat. This does not mean, however, that it can be concluded that the work efficiency is higher in an irreversible case compared to a reversible case. The concept of work efficiency applies only to reversible cycles and not to irreversible, non-cyclical processes. In fact, as with heat efficiency, the higher the work efficiency, the better. Higher work efficiency means that when a reversible isobaric-isochoric cycle runs in reverse (a positive cycle), with the amount of work input from the low-pressure work reservoir fixed (keeping the amount of work recovered constant), more work is output from the high-pressure work reservoir (more net work is converted from heat). Moreover, for purely irreversible processes, only work can be converted to heat, and heat cannot be converted to work. In terms of heat production as a gain, the heat obtained by an irreversible process can only be converted from an equal amount of work, whereas the heat obtained by a reversible cycle can be absorbed from the external environment in addition to the portion that is converted from the net work.

For a heat pump, the work input to the system can be divided into two parts, one part of which is converted into (net) heat, and the other part is then output from the system. The latter is inevitable in keeping the thermodynamic cycle running, and it can be recovered. This is why the performance index of heat pumps, the coefficient of performance (COP), usually only takes into account the part of the (net) work that is converted to heat. However, in a practical application, all the work participating in the cycle inevitably generates mechanical losses during the delivery or the recovery. Therefore, under the condition that the part of the work that is converted to heat is fixed, the less work remaining to participate in the cycle, the better, and this can be evaluated using the concept of work efficiency. Specifically, for a heat pump, the higher the work efficiency, the less the work that needs to be recovered and the better the heat pump performance. Work efficiency can be used together with the COP to achieve a more comprehensive performance evaluation of a heat pump.

Table 1 provides a comparison of the newly established laws of the reversible heat–work conversion cycle with the conventional version.

## 4. Conclusions

The Clausius statement of the second law of thermodynamics says that heat can never pass from a cold to a warm body without some other change. Then, it is generally considered that the second law of thermodynamics is a rule about process irreversibility. However, in fact, what Clausius originally referred to as the second law of thermodynamics was the theorem of the equivalence of two kinds of transformations, i.e., the transformation of work to heat and the transformation of heat at a high temperature to a low temperature in a reversible cycle, which was explicitly mentioned in his memoirs. The TET is a law about reversible thermodynamics, and the Carnot efficiency was determined and the concept of entropy was discovered based on this law. The effects of irreversibility were only formulated as corollaries of the TET.

A TET in a reversible work-to-heat cycle is proposed, which is symmetrical to Clausius’s TET. It points out that the two transformations, i.e., the transformation of heat to work and the transformation of work from high pressure to low pressure, should be equivalent. The proposed symmetric TET leads to the other half of the cyclic laws of the mutual conversion between heat and work. A performance index of work efficiency symmetrical to the Carnot efficiency is proposed, and a theorem symmetrical to Carnot’s theorem is also presented. Similar to Carnot’s theorem, the work efficiency of a reversible isobaric-isochoric cycle is only dependent on the pressures of two work reservoirs. Clausius’s TET led to the discovery of the concept of entropy, while the proposed symmetric TET offers a new physical meaning for volume. Considering the symmetry between entropy and volume, volume can be called “work entropy” just as entropy was called “heat volume” by von Oettingen.

## Figures and Tables

**Table 1:** The symmetrical framework of the laws of the reversible heat–work conversion cycle.

	Heat-to-Work	Work-to-Heat
**Fundamental principle**	Heat can never pass from a cold to a warm body without some other change	Volumetric work can never pass from low pressure to high pressure without some other change
**Second law**	Theorem of equivalence between the transformation of work to heat and the transformation of heat from high to low temperatures	Theorem of equivalence between the transformation of heat to work and the transformation of work from high to low pressures
**Basic cycle**	Carnot cycle	Isobaric-isochoric cycle
**Cycle theorem**	The heat efficiency of a reversible Carnot cycle is only dependent on the temperatures of two heat reservoirs (Carnot’s theorem)	The work efficiency of a reversible isobaric-isochoric cycle is only dependent on the pressures of two work reservoirs
**M** **aximum efficiency**	Carnot heat efficiency: $\eta_{C}=1-\frac{T_{2}}{T_{1}}$	Work efficiency of a reversible isobaric-isochoric cycle: $\eta_{W}=1-\frac{P_{2}}{P_{1}}$
**Characteristic variable**	Entropy: $dS=\frac{\delta Q_{\mathrm{rev}}}{T}$	Volume: $dV=-\frac{\delta W_{\mathrm{rev}}}{P}$

## Data Availability

Data are contained within the article.
